# The efficacy of tranexamic acid in reducing intraoperative bleeding during transurethral resection of the prostate for benign prostatic hyperplasia (BPH): a systematic review and meta-analysis of randomized controlled trials

**DOI:** 10.7717/peerj.20780

**Published:** 2026-02-11

**Authors:** Yanwei Zhang, Ming Liu, Fengze Sun, Bin Wang, Yicheng Guo, Yuchen Qian, Jitao Wu

**Affiliations:** 1 Binzhou Medical University, Second Clinical Medical College, Yan Tai, Shan Dong, China; 2 Yantai Yuhuangding Hospital, Department of Urology, Yan Tai, Shan Dong, China

**Keywords:** Tranexamic acid, Transurethral resection of prostate, Perioperative bleeding, Meta-analysis, Urological surgery, Hemostatic efficacy

## Abstract

**Background:**

Benign prostatic hyperplasia (BPH) is a common urinary system disease in elderly men, and transurethral resection of the prostate (TURP) is the gold standard for treating BPH. However, this surgery often leads to intraoperative and postoperative bleeding. Tranexamic acid (TXA) is an antifibrinolytic drug commonly used for hemostasis. This study aims to investigate the hemostatic effect of tranexamic acid in TURP surgery.

**Aim:**

Evaluate the efficacy of tranexamic acid in TURP surgery.

**Methods:**

We systematically searched the PubMed, Embase, EBSCO, Cochrane Library, and Web of Science databases for randomized controlled trials (RCTs) on TXA treatment for bleeding during transurethral resection of the prostate (TURP), published up to December 2025. Data analysis and management were performed using Review Manager version 5.3.

**Result:**

After applying the predefined inclusion and exclusion criteria, a total of eight studies involving 611 patients were included in our meta-analysis. The results of our analysis indicated that the TXA group significantly outperformed the control group in three outcomes: intraoperative blood loss (*P* = 0.04), postoperative 24-hour hemoglobin levels (*P* < 0.001), and postoperative 24-hour hemoglobin difference (*P* = 0.02). However, no significant differences were observed between the TXA and control groups regarding surgical time (*P* = 0.28) and length of hospital stay (*P* = 0.08).

**Conclusions:**

Compared to the control group, TXA significantly reduces intraoperative and postoperative bleeding in TURP surgery, making it valuable for anemic patients and in reducing surgical complications.

## Introduction

Benign prostatic hyperplasia (BPH) is one of the most common benign diseases affecting aging men, and its prevalence increases markedly with age, reaching more than 50% in men over 60 years old and up to 80% in those over 80 years old. Patients with BPH often present with lower urinary tract symptoms (LUTS) (Lloyd, Marks & Ricke, 2019; Langan, 2019), including urinary urgency, increased frequency, weak urinary stream, nocturia, or urinary retention. In severe cases (Montorsi et al., 2023), it can lead to hydronephrosis (Sutaria & Staskin, 2000). For patients with moderate to severe symptoms who respond poorly to medical therapy, transurethral resection of the prostate (TURP) remains the gold-standard surgical treatment recommended by international guidelines (Gilling, 2014; Huang et al., 2019; Lokeshwar et al., 2019). However, this procedure is associated with certain complications, particularly intraoperative and postoperative bleeding. The vascular-rich nature of the hyperplastic prostate tissue increases the risk of bleeding (McNeal, 1988). Studies have shown that the incidence of significant blood loss requiring transfusion during TURP ranges from 0.4% to 7.1% (Kloping, Yogiswara & Azmi, 2022; Mina & Garcia-Perdomo, 2018). Additionally, the high fibrinolytic activity caused by the release of urokinase from prostate tissue is also considered a key factor contributing to increased bleeding (Eraky et al., 2024), as urine and urothelial cells contain high levels of plasminogen activators that stimulate the fibrinolytic system. Based on this mechanism, researchers have evaluated the role of various antifibrinolytic agents in reducing blood loss during TURP, among antifibrinolytic drugs, tranexamic acid (TXA) (Cai et al., 2020; Zehtabchi et al., 2014) is the most widely used. It works by competitively inhibiting the activation of plasminogen to plasmin, thereby blocking fibrinolysis. Systematic reviews and meta-analyses have shown that TXA is highly effective in reducing blood loss and the need for transfusions in cardiac, orthopedic, gynecological, and transplant surgeries (Chen et al., 2016; Bailey, 2023; Cheriyan et al., 2015; Massicotte et al., 2011; Topsoee et al., 2016). However, its efficacy and safety in prostate surgery have not yet been fully established. Therefore, we conducted a systematic review and meta-analysis of randomized controlled trials (RCTs) to evaluate whether TXA can effectively reduce perioperative bleeding in patients undergoing TURP for BPH. This study aims to provide updated, evidence-based guidance for the clinical application of tranexamic acid in BPH surgery.

## Materials and Methods

### Search strategy

We conducted a literature search according to our inclusion and exclusion criteria, and reported the results of the meta-analysis in accordance with the Preferred Reporting Items for Systematic Reviews and Meta-Analyses (PRISMA) guidelines (Moher et al., 2009) (Table S1). As of December 2025, we have retrieved all the published English-language articles on the efficacy of TXA in reducing blood loss during transurethral resection of the prostate (TURP) from major databases, including PubMed, Embase, EBSCO, Cochrane Library, and Web of Science. The keywords and search string we used are as follows: “((Tranexamic Acid) OR (AMCHA) OR (trans-4-(Aminomethyl) cyclohexanecarboxylic Acid) OR (t-AMCHA) OR (AMCA) OR (Anvitoff) OR (Cyklokapron) OR (Ugurol) OR (KABI 2161)) AND ((Transurethral Resection of Prostate) OR (TURP) OR (VLAP) OR (TUVP) OR (Prostate Transurethral Resection) OR (Prostate Transurethral Resections) OR (Transurethral Prostate Resection))”. This systematic review and meta-analysis has been published on the PROSPERO international prospective register of systematic reviews, and the registration ID is CRD42024616139.

Prior to PROSPERO registration submission, only title/abstract screening was conducted to rapidly exclude clearly irrelevant records (*e.g.*, studies with mismatched disease types, non-interventional research). This procedure did not involve full-text assessment or inclusion decisions. As a technical preparatory step in the literature screening workflow, it aimed to reduce subsequent workload while preserving the prospective design integrity of the core protocol (*e.g.*, final inclusion criteria and analytical plans were executed post-registration). Full-text review, data extraction, and other critical processes were implemented after PROSPERO registration approval (Registration ID: CRD42024616139), aligning with prospective registration standards. This approach adheres to systematic review methodology, with transparency ensured through timestamp documentation in the PRISMA flow diagram.

Literature retrieval and screening were independently performed by two reviewers:

First reviewer: Yanwei Zhang (Lead investigator; responsible for search strategy development and preliminary screening). Second reviewer: Ming Liu(Data analyst; responsible for cross-verification and full-text screening).

Roles were assigned based on core responsibilities, with Yanwei Zhang (first author) leading the retrieval process and Ming Liu (co-first author) overseeing quality control, consistent with international authorship contribution standards for systematic reviews.

Following dual independent screening, the majority of discrepancies were resolved through daily reconciliation meetings with reference to the predefined Screening Operational Protocol. For persistent disagreements involving eligibility criteria disputes or data integrity conflicts, the quality controller (Bin Wang) arbitrated final decisions based on the original study protocol and PRISMA guidelines.

### Study selection

**Figure 1:**
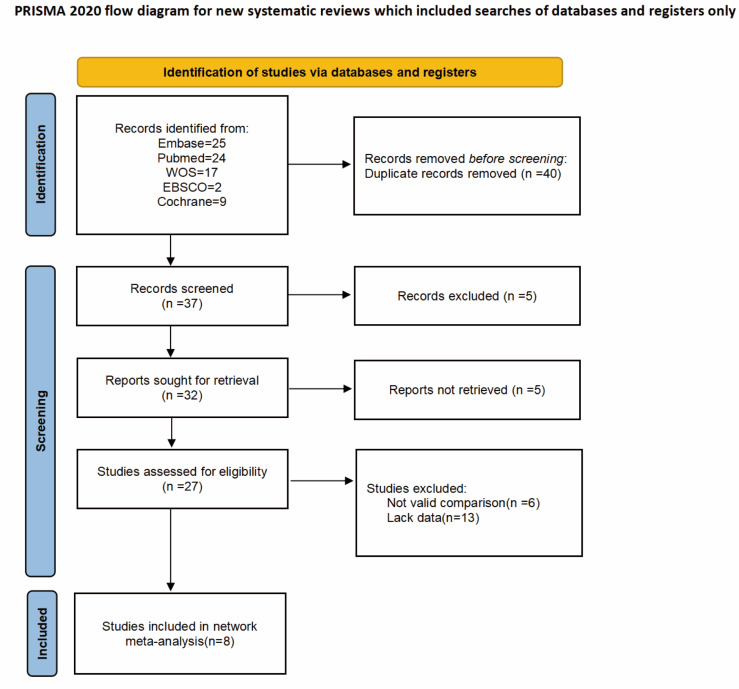
PRISMA flow diagram.

The potential eligible studies were first screened by title and abstract, followed by full-text review. Studies that met the following criteria were included: (1) Randomized controlled trials (RCTs); (2) BPH patients undergoing monopolar or bipolar TURP treatment; (3) Studies comparing tranexamic acid intervention with placebo or standard treatment; (4) Studies reporting at least one of the following outcomes: intraoperative blood loss, 24-hour hemoglobin changes, length of hospital stay, or surgical time. Articles without full-text access or incomplete data were excluded. Ultimately (Fig. 1), eight studies were included (Diab et al., 2024; Gupta et al., 2021; Jendoubi et al., 2017; Karkhanei et al., 2019; Meng et al., 2019; Samir et al., 2021; Tawfick et al., 2022; Vanderbruggen et al., 2023).

### Quality assessment

**Figure 2:**
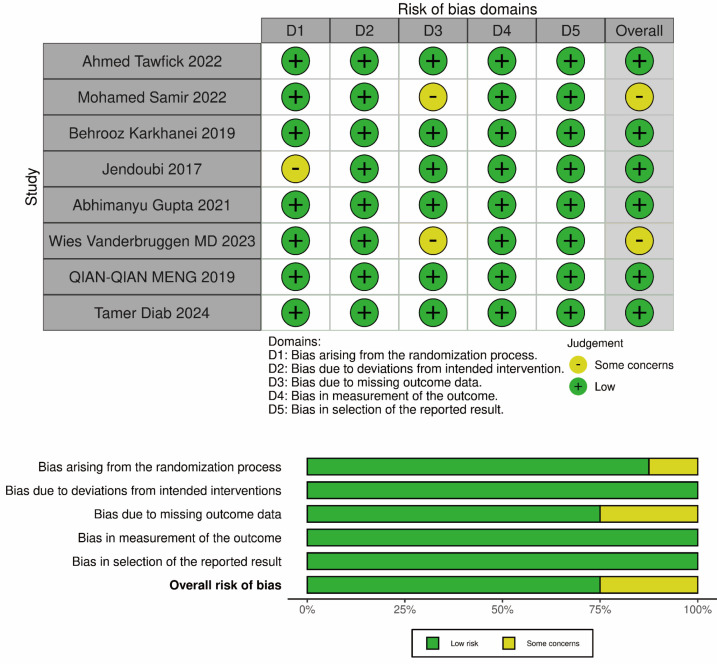
The assessment of risk of bias (RoB).

The risk of bias in randomized controlled trials (RCTs) was analyzed using the Cochrane Risk of Bias (RoB) tool (Fig. 2). The key domains assessed included the randomization process, deviations from the intended interventions, missing outcomes, outcome measurement methods, and selection of reported results. Each domain was rated as “low risk,” “some concerns,” or “high risk” based on the completeness of the description in the methods section of the trials. Any disagreements or discrepancies were resolved through discussion among the researchers.

### Data extraction

The researchers extracted content from the articles and created data tables (Table 1). The extracted information included the authors, publication year, sample characteristics (such as gender, prostate weight), intervention details, and study outcomes.

**Table 1:** Characteristics of included studies.

Study	Age: intervention/control	Prostate size intervention/ control	N. patients: intervention/ control	Intervention
Tawfick et al. (2022)	69.36 ± 7.88/68.88 ± 7.82	70.28 ± 9.17/68.88 ± 9.27	25/25	Group A received 0.1% TXA 1,000 mg (10 mL) in one L of irrigation solution of sterile wash (glycine) during surgery
Diab et al., 2024	67.3 ± 9.71/71.13 ± 9.06	64.5 ± 10.44/68.1 ± 11.47	30/30	Group I received an intraprostatic injection of one g of TXA (Cyklokapron) dissolved in 50 mL of 0.9% saline at multiple sites
Samir et al., 2021	64.66 ± 5.87/65.75 ± 5.48	108.32 ± 16.64/107.09 ± 16.21	95/91	Group A underwent B-TURP and received TXA as an intravenous loading dose of 50 mg/kg over 20 min before induction of anaesthesia followed by a maintenance infusion of 5 mg/kg/h untilresection was completed.
Karkhanei et al. (2019)	66.43 ± 7.86/69.63 ± 9.67	36.62 ± 16.29/39.14 ± 19.9	35/35	The study group participants received 500 mg TXA in 500 ml ringer serum (concentration of 1 mg/ml, infusion rate 100 mg/min, maximum dose of 15 mg/kg)
Jendoubi et al. (2017)	67.31 ± 7.72/71.13 ± 9.06	47.76 ± 16.41/59.07 ± 30.62	30/30	TXA: bolus of 10 mg/kg at the induction of anesthesia followed by infusion of 1 mg/kg/h intraoperatively and for 24 h postoperatively
Gupta et al. (2021)	68.20 ± 8.49/66.51 ± 9.38	56.87 ± 14.91/51.2 ± 17.03	35/35	The study group (1) received IV TXA 500 mg after induction of anesthesia and 500 mg in each irrigation fluid bottle (dual mode) and the control group (2) received none.
Vanderbruggen et al. (2023)	65.6/66.6	52.3/55.7	31/34	The TXA group (31 patients) received a TXA intravenous loading dose of 10 mg/kg over 30 min before induction, followed by a maintenance dose of 5 mg/kg/h over 12 h
Meng et al. (2019)	71.4 ± 5.4/70.7 ± 8.5	73.3 ± 8.3/66.6 ± 3.9	30/30	The TXA group was intravenously administered with one g TXA

### Statistical analysis

We used Review Manager 5.3 for data processing. For continuous variables, the mean difference (MD) and 95% confidence interval (CI) were used for comparison, and the results were expressed as mean ± standard deviation (SD). We assessed heterogeneity using *p*-values and I^2^, Heterogeneity was quantified using Cochran’s *Q* test and Higgins’ I^2^ statistic as described by Higgins et al. (2003). If the *p*-value < 0.05 and I^2^ > 50%, we used a random-effects model. If the *p*-value >0.05 and I^2^ <  50%, indicating low heterogeneity, we applied a fixed-effects model. Additionally, if the final result showed *p* < 0.05, it indicated that the difference was statistically significant.

## Results

### Postoperative 24-hour hemoglobin difference

**Figure 3:**
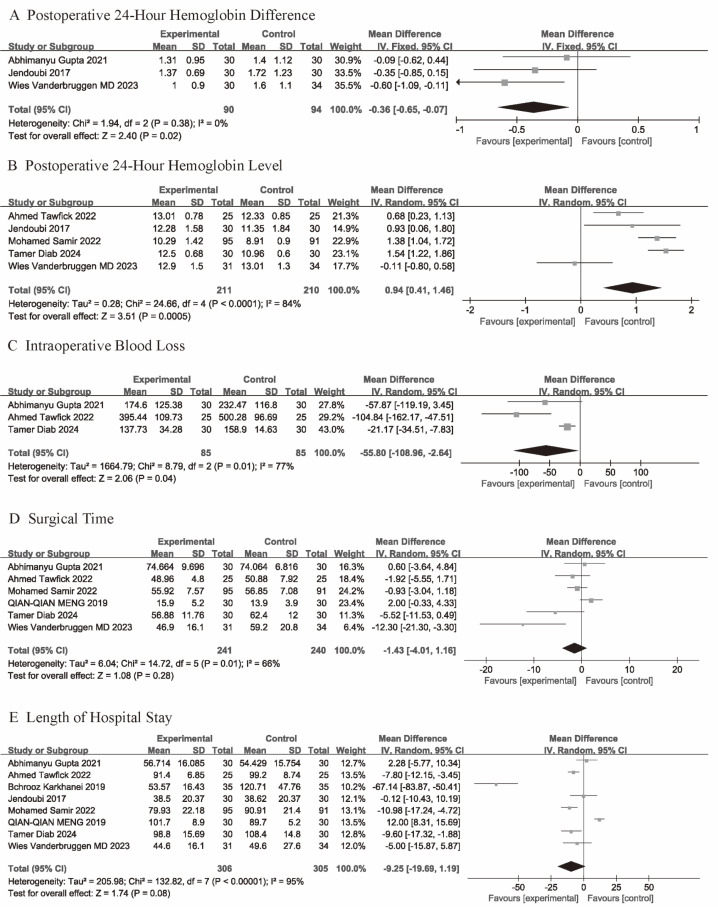
(A–E) Forest plots showing the pooled results.

Three studies provided data on the 24-hour postoperative hemoglobin difference from 185 participants (TXA = 91, control = 94). Given the low heterogeneity between the studies (*p* = 0.38, I^2^ = 0%), a fixed-effects model was used for the assessment (Fig. 3A). The analysis results showed that the TXA group had a significantly lower 24-hour postoperative hemoglobin difference (MD = −0.36; 95% CI [−0.65 to −0.07]; *P* = 0.02).

### Postoperative 24-hour hemoglobin level

Five studies provided data on postoperative 24-hour hemoglobin levels. Due to considerable heterogeneity between the studies (*p* < 0.001; I^2^ = 84%), a random-effects model was applied. The results showed that the TXA group had significantly higher hemoglobin levels compared to the control group (Fig. 3B) (MD = 0.94; 95% CI [0.41–1.46]; *p* < 0.001).

### Intraoperative blood loss

Five studies provided data on intraoperative blood loss. Due to considerable heterogeneity between the studies (*p* = 0.01; I^2^ = 77%), a random-effects model was applied. The results showed that the TXA group had significantly less intraoperative blood loss compared to the control group (Fig. 3C) (MD = −55.8; 95% CI [−108.96 to −2.64]; *p* = 0.04).

### Surgical time

Six studies provided data on surgical time. Due to high heterogeneity between the studies (*p* = 0.01; I^2^ = 66%), a random-effects model was used to calculate the mean difference (MD) and 95% confidence interval (CI). The analysis showed no significant difference in surgical time between the TXA group and the control group (MD = −1.43; 95% CI [−4.01 to 1.16]; *p* = 0.28) (Fig. 3D).

### Length of hospital stay

Data on Length of hospital stay were provided by all studies. Due to considerable heterogeneity between the studies (*p* < 0.001; I^2^ = 95%) (*p* = 0.01; I^2^ = 66%), a random-effects model was applied. The results showed that there was no significant difference between the TXA group and the control group in terms of hospital stay duration (Fig. 3E) (MD = −9.25; 95% CI [−19.69–1.19]; *p* = 0.08).

## Discussion

Tranexamic acid (TXA) is a synthetic lysine analogue that inhibits the conversion of plasminogen to plasmin and suppresses fibrinolysis (Franchini, Focosi & Mannucci, 2024). During transurethral resection of the prostate (TURP), the release of urokinase and other plasminogen activators from prostatic tissue can trigger fibrinolytic activity and contribute to persistent bleeding intra- and post-operatively. Based on this biological mechanism, the application of TXA in TURP has a clear theoretical rationale (Kavanagh, Jack & Lawrentschuk, 2011). Our meta-analysis demonstrated that TXA significantly reduced intraoperative blood loss, maintained higher postoperative hemoglobin levels, and decreased 24-hour hemoglobin decline compared with control interventions. These findings suggest that antifibrinolytic therapy plays a meaningful role in minimizing perioperative bleeding during TURP.

### Cross-disciplinary comparison

When compared across surgical and trauma disciplines, the efficacy of TXA appears context-dependent. Tang et al. (2023) reported that perioperative TXA in calcaneal fracture surgery significantly reduced postoperative 24-hour blood loss (SMD −0.99, 95% CI [−1.38 to −0.61]; *p* < 0.001; I^2^ = 0%) and improved hemoglobin and hematocrit levels (SMD 0.77 and 0.92, respectively), while lowering wound complication rates without increasing thrombotic events. Similarly, Buheiri et al. (2025) found that systemic TXA in breast surgery reduced postoperative hematoma (OR 0.43, 95% CI [0.23–0.81]) and seroma (OR 0.57, 95% CI [0.35–0.92]) formation and decreased drainage volume by a mean of 132 mL (95% CI [44–220] mL), again without evidence of increased thromboembolism.

Beyond elective surgery, Song et al. (2024) demonstrated in a meta-analysis of traumatic brain injury (TBI) that TXA reduced mortality (RR 0.92, 95% CI [0.85–1.00]; *p* = 0.05) and hemorrhage growth (RR 0.78, 95% CI [0.62–0.97]; *p* = 0.03), while not affecting rates of neurosurgical intervention, seizures, or pulmonary embolism. These consistent signals from orthopedic, neurotrauma, and now urologic settings support the view that TXA’s antifibrinolytic mechanism may exert its greatest benefit in controlled bleeding environments with direct visualization—conditions that closely resemble TURP.

Conversely, in uncontrolled bleeding scenarios such as acute gastrointestinal hemorrhage, large-scale trials like HALT-IT failed to show comparable benefits and even indicated possible increases in adverse events. This discrepancy underscores that TXA efficacy depends on the controllability and focal nature of the bleeding field: TURP resembles orthopedic or reconstructive surgery more closely than diffuse mucosal hemorrhage (O’Donnell et al., 2024).

### Interpretation in the context of TURP

Despite the clear hemostatic advantages, TXA did not shorten operative time or hospital stay in our analysis. Across all included trials, the pooled mean difference in hospital stay duration (six studies) was not significant (MD = −1.43 days; 95% CI [−4.01 to 1.16]; *p* = 0.28), with moderate heterogeneity (I^2^ = 66%, *p* = 0.01). Likewise, although all studies reported surgical time, the pooled analysis using a random-effects model—necessitated by very high heterogeneity (I^2^ = 95%, *p* < 0.00001)—showed no significant difference in operative duration between TXA and control groups.

These neutral findings parallel those of Tang et al. (2023) and Buheiri et al. (2025), who observed improved hemostasis but no measurable acceleration in postoperative recovery. In the context of TURP, operative duration is predominantly driven by prostate size, surgeon experience, and resection technology, whereas hospitalization length is influenced by standardized postoperative care pathways, such as catheter management, monitoring for electrolyte disturbances, and management of comorbidities. Thus, although TXA conserves perioperative blood and stabilizes hemoglobin, this does not necessarily translate into shorter procedures or earlier discharge.

### Heterogeneity and safety

Heterogeneity among our included TURP studies varied by outcome. For 24-hour hemoglobin difference, heterogeneity was negligible (I^2^ = 0%, *p* = 0.38), suggesting a robust and consistent effect of TXA in attenuating early postoperative hemoglobin drop. In contrast, 24-hour hemoglobin levels (I^2^ = 84%, *p* < 0.0001), intraoperative blood loss (I^2^ = 77%, *p* = 0.01), hospital stay (I^2^ = 66%, *p* = 0.01), and surgical time (I^2^ = 95%, *p* < 0.00001) all showed substantial heterogeneity. These patterns likely reflect differences in TXA dose, timing (single bolus *vs* repeated administration), route (intravenous *vs* intravesical irrigation), anesthetic techniques, resection technology (monopolar *vs* bipolar TURP), and variability in perioperative fluid management and transfusion thresholds. Similar between-study variability has been noted in trauma and orthopedic TXA literature, emphasizing the importance of standardized dosing and reporting.

Regarding safety, no major TXA-related complications were consistently reported across the TURP trials. This aligns with data from TBI and breast surgery, where TXA did not significantly increase the risk of pulmonary embolism, seizures, or microvascular thrombosis (Song et al., 2024; Buheiri et al., 2025). Nevertheless, pharmacovigilance remains necessary, particularly in patients with pre-existing cardiovascular disease, renal impairment, or a high baseline thrombotic risk, in whom systemic accumulation or concurrent prothrombotic factors could theoretically increase adverse events.

### Limitations and future perspectives

Our analysis is limited by the modest sample size of existing RCTs, heterogeneity of dosing regimens, lack of standardized adverse-event reporting, and absence of long-term follow-up. Similar methodological issues were highlighted in both orthopedic and trauma meta-analyses. Future multicenter RCTs should adopt harmonized dosing strategies and systematically evaluate thromboembolic endpoints.

Clinically, TXA should be viewed as an adjunctive hemostatic agent rather than a determinant of surgical efficiency. It is particularly valuable in elderly or anemic patients vulnerable to hemodynamic instability. Future research should explore head-to-head comparisons between intravenous and topical routes, identify subgroups with high fibrinolytic activity or large prostates, and integrate coagulation biomarkers to elucidate mechanisms. Long-term surveillance is essential to confirm safety and define the optimal balance of efficacy and risk across surgical fields.

## Conclusion

This meta-analysis provides evidence for the application of tranexamic acid (TXA) in patients with benign prostatic hyperplasia (BPH) undergoing transurethral resection of the prostate (TURP). We found that TXA has a positive effect in reducing blood loss and lowering hemoglobin differences. However, no significant effect of TXA was found in reducing surgical time or length of hospital stay.

##  Supplemental Information

10.7717/peerj.20780/supp-1 Supplemental Information 1 PRISMA checklist

10.7717/peerj.20780/supp-2 Supplemental Information 2 PRISMA checklist

## Additional Information and Declarations

### Data Availability

The following information was supplied regarding data availability: The raw data is available in the Supplemental Files.
